# Visceral Fat Dysfunctions in the Rat Social Isolation Model of Psychosis

**DOI:** 10.3389/fphar.2017.00787

**Published:** 2017-11-08

**Authors:** Stefania Schiavone, Giulia M. Camerino, Emanuela Mhillaj, Margherita Zotti, Marilena Colaianna, Angelo De Giorgi, Antonello Trotta, Francesco P. Cantatore, Elena Conte, Maria Bove, Paolo Tucci, Maria G. Morgese, Luigia Trabace

**Affiliations:** ^1^Department of Clinical and Experimental Medicine, University of Foggia, Foggia, Italy; ^2^Department of Pharmacy and Drug Sciences, University of Bari Aldo Moro, Bari, Italy; ^3^Department of Physiology and Pharmacology, Sapienza Università di Roma, Rome, Italy; ^4^Dual Diagnosis Unit, Azienda Sanitaria Locale della Provincia di Foggia, Foggia, Italy; ^5^Rheumatology Unit, Foggia City Hospital “Ospedali Riuniti”, Foggia, Italy; ^6^Department of Medical and Surgical Sciences, University of Foggia, Foggia, Italy

**Keywords:** social isolation, psychosis, oxidative stress, adipose tissue, antioxidant enzymes, NADPH oxidase

## Abstract

Medication with neuroleptics has been associated with adipose tissue dysfunctions and, in particular, with increased visceral fat amount. However, several studies suggested that antipsychotic treatment might not be the main responsible of fat mass accumulation, as this has been also described in not treated psychotic patients. One of the most used “drug-free” rodent models of psychosis is the social isolation rearing of young adult rats, which provides a non-pharmacologic method of inducing long-term alterations reminiscent of symptoms seen in psychotic patients. Recent data highlighted a crucial role of redox imbalance in adipose tissue dysfunctions, in terms of decreased antioxidant defense and increased reactive oxygen species (ROS). Here, we investigated possible oxidative stress-related biomolecular alterations associated with visceral fat increase in 7 week isolated rats. To this purpose, we quantified total and visceral fat amount by using dual-energy X-ray (DEXA) absorptiometry. On visceral fat, we analyzed the expression of specific ROS-producer genes (*Nox1, Nox4, Hmox-1*), antioxidant enzymes (*Prdx1* and *Ucp-1*) and oxidative stress-induced damage markers (*Cidea, Slc2a4*, and *Acacb*). The impact of oxidative stress on beta3-adrenergic receptors (*Adrb3*), at both mRNA and protein level, was also assessed. We found that 7 weeks of social isolation induced an increase in total and visceral fat, associated with a decrease in *Prdx1* (mRNA and protein) as well as *Ucp-1* mRNA levels and an enhanced expression of *Nox1* (mRNA and protein) and *Hmox-1* mRNA. No differences were detected in *Nox4* mRNA levels between grouped and isolated animals. Elevations in *Cidea*, *Slc2a4*, and *Acacb* expression in visceral fat of isolated animals accounted for oxidative stress-related damage in this tissue, further associated with a significant increase in *Adrb3* mRNA and protein. Our results provide a novel understanding of the pathological link existing among psychosocial stress-induced psychosis, adipose tissue dysfunctions and redox imbalance, opening new therapeutic perspectives for the treatment of alterations in peripheral tissues associated with this mental disorder.

## Introduction

### Psychosis and Adipose Tissue Dysfunctions

Among psychiatric diseases, psychosis represents one of the most impacting cause of disability (Vigo et al., 2016), with a consequent increased international trend in antipsychotic prescription (Verdoux et al., 2010). Both typical neuroleptics (such as chlorpromazine and haloperidol) and second-generation anti-psychotics (including clozapine, olanzapine, risperidone, and aripiprazole) have been widely shown to be associated with weight gain (Medici et al., 2016), adipose tissue dysfunctions and body mass index (BMI) alterations (Konarzewska et al., 2014), most likely via impaired adipokine and pro-inflammatory cytokine pathways (Kucerova et al., 2015), as well as increased food intake, delayed satiety signaling, insulin resistance and glucose dysregulation (Deng, 2013; Manu et al., 2015). However, some studies have described weight elevation, adipose tissue dysfunctions, as well as other metabolic alterations, also in drug-free and drug-naïve psychotic patients (Ryan et al., 2003, 2004; Spelman et al., 2007), suggesting that antipsychotic medication might not be the main responsible of these disturbances. Indeed, increased visceral fat distribution has been described as one of the most important pathogenetic process, accounting for weight impairment and enhanced risk of metabolic syndrome development in untreated psychotic patients (Thakore et al., 2002).

### The Social Isolation Model in Rats to Mimic the “Drug-Naïve” Status

On the other hand, several studies have focused on first psychotic episode subjects to investigate whether drug-free patients were more likely prone to develop a metabolic disorder than general population (Britvic et al., 2013; Misiak et al., 2016). Unfortunately, only few studies from the pre-medication era are actually available and some biases in the methodological aspects, as well as the use of different diagnostic criteria, make their interpretation arduous. In particular, some studies have included, as “drug-free,” patients medicated for a short period of time (Mane et al., 2009). Hence, as the status of “drug-free” or “drug-naïve” is difficult to be found in a psychotic patient, studies on non-pharmacologic animal models of psychosis are particularly useful. One of the most used drug-free rodent model of psychosis is the social isolation rearing of young adult rats (Weiss and Feldon, 2001; Leng et al., 2004), which provides a non-pharmacologic method of inducing long-term alterations reminiscent of several symptoms seen in psychotic patients (King et al., 2009), including hyperreactivity to novel environments and cognitive impairment (Geyer and Ellenbroek, 2003; Lapiz et al., 2003). Indeed, social isolation after weaning represents a stressful condition for rats, normally living in a gregarious environment. When reared under isolation, they show a pattern of behavioral and neurobiological alterations, which are thought to be a product of a prolonged stress (Fone and Porkess, 2008). Although the underlying pathological mechanisms are poorly understood, social deprivation of rat pups during a critical period, such as from weaning through adulthood, appears as a strong psychosocial stress, which is thought to contribute to the etiology of mental disorders, such as psychiatric conditions in humans (Schiavone et al., 2013). Together with these symptoms, accumulating evidence suggests that deprivation of social interactions is significantly associated with several metabolic disturbances, including hypertension, cardiovascular dysfunctions, and adipose tissue dysfunctions (Bartolomucci et al., 2009).

### The Role of Redox Imbalance in Adipose Tissue Dysfunctions

Recent data highlighted a crucial role of redox imbalance, defined as a disequilibrium between free radical production and degradation, in adipose tissue dysfunctions (Jankovic et al., 2015b). In particular, both mitochondria and other reactive oxygen species (ROS) producers, such as the NADPH oxidase NOX enzymes or the nitric oxide synthase system have been described as key redox modulators in the adipose tissue (Daiber, 2010; Koh et al., 2010). On the other hand, decrease in antioxidant defense has been shown to be a crucial contributor to the onset and progression of adipocyte dysfunctions (Curtis et al., 2010; Jankovic et al., 2016; Yadav et al., 2016). We have previously demonstrated an increased expression of the NADPH oxidase NOX2 enzyme in the central nervous system (CNS) of socially isolated rats (Schiavone et al., 2009, 2012, 2017).

Thus, the main aim of the present study was to investigate the existence of a possible pathogenetic link between oxidative stress-related biomolecular alterations and white adipose tissue dysfunctions in a “drug-free” rat model of psychosis induced by 7 weeks of social isolation. To this purpose, we compared the amount of total and visceral fat in animals reared either in social isolation (7 weeks) or in social groups, by using dual-energy X-ray (DEXA) absorptiometry. mRNA levels of specific genes encoding for ROS producer and antioxidant enzymes, as well as for oxidative stress-induced damage in the adipose tissue, were also assessed. As secondary objective, we aimed to assess if alterations of the redox state might impact the expression of Adrb3 in visceral fat, given their crucial role in the pathophysiology of adipose tissue.

## Materials and Methods

### Animals

Adult male and female Wistar rats (Envigo, San Pietro al Natisone, Italy) weighting 250–280 g were housed at constant room temperature (22 ± 1°C) and relative humidity (55 ± 5%) under a 12 h light/dark cycle (lights on from 7:00 AM to 7:00 PM) for at least 7 days before the experiments. Food and water were available *ad libitum*. Procedures involving animals and their care were conducted in conformity with the institutional guidelines of the Italian Ministry of Health (D.L. 26/2014; protocol authorization number: 171/2017 PR) the Guide for the Care and Use of Laboratory Animals: Eight Edition, the Guide for the Care and Use of Mammals in Neuroscience and Behavioral Research (National Research Council, 2004), the Directive 2010/63/EU of the European Parliament and of the Council of 22 September 2010 on the protection of animals used for scientific purposes. All procedures involving animals were conducted in accordance to ARRIVE guidelines. Animal welfare was daily monitored through the entire period of experimental procedures. No signs of distress were evidenced, anyway all efforts were made to minimize the number of animals used and their suffering.

### Social Isolation Protocol

The social isolation protocol was performed as previously described (Schiavone et al., 2016). Briefly, one male and two females were housed together for mating (Leng et al., 2004). At weaning (postnatal day 21), pups were separated from their mothers and reared either as isolated rats (ISO; one rat per cage) or reared in group as control rats (GRP; three to four rats per cage). To avoid a litter effect, each litter contributed only one male subject to the GRP and one male subject to the ISO. All animals were reared in Plexiglas cages (ISO: 40.0 cm × 27.0 cm × 20.0 cm; GRP: 59.0 cm × 38.5 cm × 20.0 cm). ISO and GRP rats were housed in the same room, so that isolated rats maintained a visual, auditory, and olfactory contact with the other animals. Rats were disturbed only for cleaning purposes (changing the cage once a week for ISO and GRP) and for the weekly body weight measurement, throughout the whole experiment. At the end of the social isolation protocol, the assessment of behavior was performed by evaluating the locomotor activity of control and isolated animals by the Open Field test and the discrimination ability by using the Novel Object Recognition test, as previously described (Schiavone et al., 2009, 2012). Animals exposed to the social isolation procedure described in the present work showed an increased locomotor activity and a reduction in the discrimination index with respect to rats reared in group (data not shown), further confirming what previously observed from our group (Schiavone et al., 2009, 2012). All the other experimental procedures were conducted immediately after the behavioral assessment.

### Body Weight and Food Intake Evaluation

Grouped and isolated rats were weekly weighted. Data were expressed as body weight gain, thus as the difference between initial body weight (week 1 of the social isolation period) and final body weight (week 7 of the social isolation period). Individual food intake was evaluated weekly for ISO rats, while, for GRP animals, the mean of food intake was considered. Data were expressed as feeding efficiency, indicated as g of body weight gained for g of food intake.

### Blindness of the Study

Researchers performing analysis were blind with respect to the rat rearing conditions, as it was not possible to deduce from the labeling whether an animal was isolated or not. The social isolation procedure was performed in a dedicated part of the animal facility, not accessible to the investigators during the entire period of the social isolation protocol. The blinding of the data was maintained until the analysis was terminated.

### *In Vivo* Dual Energy X-ray Absorptiometry Analysis

**FIGURE 1:**
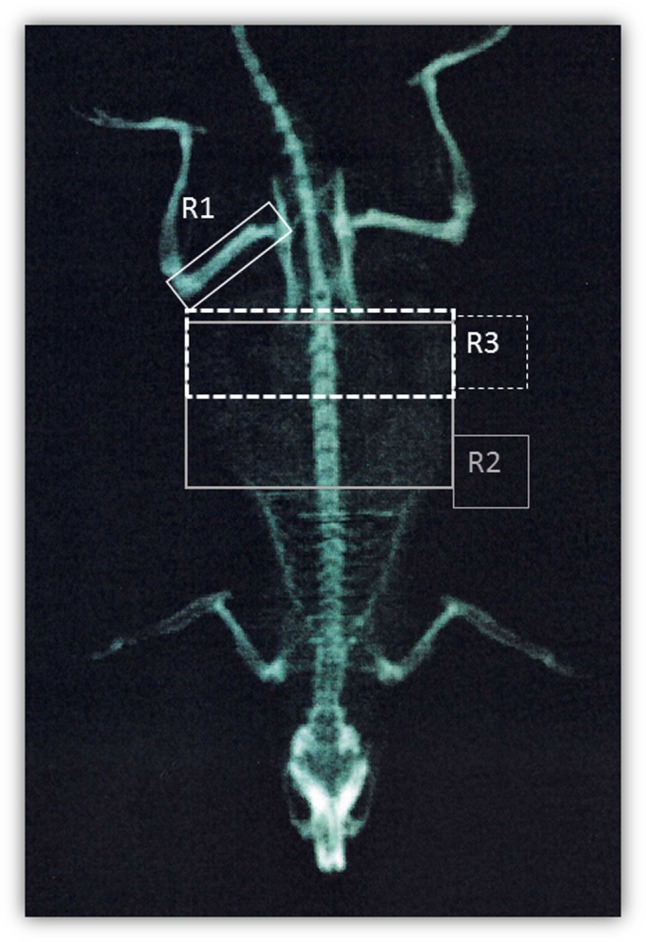
DEXA scanning. Representation of regions of interest used for DEXA quantification: right femur (R1), T9-L5 vertebrae (R2), and L1-L6 vertebrae (R3).

At the end of the social isolation period, *in vivo* dual energy X-ray absorptiometry analysis (DEXA) was performed as previously described (Schiavone et al., 2016) with a body scan densitometer (Hologic Dexa Bone Densitometer, Hologic Italia S.R.L., Rome, Italy). Briefly, before measurements, body calibration scan was performed with the Hologic phantom for small animals. Animals were positioned ventrally with the forelimbs away from the trunk to scan the whole body. The appropriate software program for small animals (DEXA; L & R Hip Software Ver. 11.1 for Windows) was used. After the scan, three regions of interest (ROI) were marked; right femur (R1), T9-L5 vertebrae (R2) and L1-L6 vertebrae (R3) (**Figure 1**). All animal images were scanned and analyzed by the same operator. Total fat mass was represented by R1+R2+R3 fat mass, while visceral fat referred to R3 fat mass, as previously described (Gerbaix et al., 2010).

### Visceral Fat Collection

Visceral fat was collected from the posterior wall of the abdominal cavity, by an area delimited in the center by the spinal structure, from one side by the abdominal wall, on the upper part by the diaphragm and in the lower part by the pelvic floor.

### Isolation of Total RNA, Reversed Transcription, and Real-Time PCR

Immediately after collection, visceral adipose tissue was frozen in liquid nitrogen and stored at -80°C until use. The qPCR experiments were conducted as previously described (Camerino et al., 2014). Briefly, 400 ng of total RNA was added to 1 μl dNTP mix 10 mM (Roche N.C. 11277049001, Switzerland) and 1 μl RandomHexamers 50 mM (Life Technologies C.N. n808-0127, United States) and incubated at 65°C for 5 min. Afterward, 4 μl 5X First Standard Buffer (Life Technologies C.N. Y02321), 2 μl 0.1 M DTT (Life Technologies C.N. Y00147) and 1 μl Recombinant RNA Ribonuclease Inhibitor 40 U/ml (Promega, C.N. N2511, United States) were added and incubated at 42°C for 2 min. One μl of Super Script II Reverse Trascriptase 200 U/ml (Life Technologies C.N. 18064-014) was added to each solution and incubated at 25°C for 10 min, at 42°C for 50 min and at 70°C for 15 min. Real-time PCR was performed in triplicate using the Applied Biosystems Real-time PCR 7500 Fast system (United States), MicroAmp Fast Optical 96-Well Reaction Plate 0.1 ml (Life Technologies C.N. 4346906) and MicroAmp Optical Adhesive Film (Life Technologies C.N. 4311971). The setup of reactions consisted of 8 ng cDNA, 0.5 μl of TaqManGeneExpression Assays (Life Technologies), 5 μl of TaqMan Universal PCR master mix No AmpErase UNG (2X) (Life Technologies C.N. 4324018) and Nuclease-Free Water not Diethylpyrocarbonate (DEPC-Treated) (Life Technologies C.N. AM9930) for a final volume of 10 μl. The following RT-TaqMan-PCR conditions were as follows: step 1: 95°C for 20 s, step 2: 95°C for 3 s and step 3: 60°C for 30 s; steps 2 and 3 were repeated 40 times. The results were compared with a relative standard curve obtained by five points of 1:4 serial dilutions. The mRNA expression of the genes was normalized to the best housekeeping gene phosphoribosyltransferase 1 (Hprt1) selected from glyceraldehyde-3-phosphate dehydrogenase (Gapdh) beta-actin (Actinb) and Hprt1 by BestKeeper and NorFinder software. TaqMan Hydrolysis primer and probe gene expression assays were obtained by Life Technologies with the following assay IDs: Uncoupling protein 1 (mitochondrial, proton carrier) (*Ucp1*) IDs: Rn00562126_m1; Cell death activator (*Cidea*) IDs: Rn04181355_m1; Solute carrier family 2 (facilitated glucose transporter), member 4 (*Slc2a4*) IDs: Rn01752377_m1; Acetyl-CoA carboxylase beta (*Acacb*) IDs: Rn00588290_m1; NADPH oxidase (*Nox1*) IDs: Rn00586652_m1; NADPH oxidase (*Nox4*) IDs: Rn00585380_m1; heme oxygenase (decycling) 1 (*Hmox1*) IDs: Rn00561387_m1; adrenergic beta 3 receptor (*Adrb3*) IDs: Rn00565393_m1 *Hprt1* IDs: Rn01527840_m1; *Actb* IDs: Rn00667869_m1 and; *Gapdh* IDs: Rn_01775763_g1. All gene expression experiments were conducted following the MIQE guideline (Bustin et al., 2009).

### Enzyme-Linked Immunosorbent Assay (ELISA)

PRDX1, NOX1 and Adrb3 protein levels in visceral fat were determined by enzyme-linked immunosorbent assay (ELISA), using commercial ELISA kits (Cloude-Clone Corporation, Houston, TX, United States). Assays were performed according to the manufacturer’s instructions. To normalize data and negate differences due to sample collection, protein concentration was determined by using the BCA (Thermo Fisher) assay kit. Each sample analysis was carried out in duplicate to avoid intra-assay variations.

8-OHdG quantification was performed by using the Highly Sensitive 8- OHdG Check Elisa Kit; (JaiCa, Japan), as previously described (Colaianna et al., 2013).

### Statistical Analysis

Data were analyzed using Graph Pad^®^ 6.0 software for Windows. Sample size for behavioral and DEXA assessment was calculated with GPower 3.1. Data were analyzed by unpaired Student’s *t*-test or by two-way ANOVA for repeated measures for feeding efficiency data. Data were normally distributed, however in case of non-normal distribution, as indicated by *F*-test, Welch’s correction was applied. For all tests, a *p* < 0.05 was considered statistically significant. Results are expressed as means ± mean standard error (SEM) or as percentage of increase compared to controls.

## Results

### Increase of Total and Visceral Fat after 7 Weeks of Social Isolation

**FIGURE 2:**
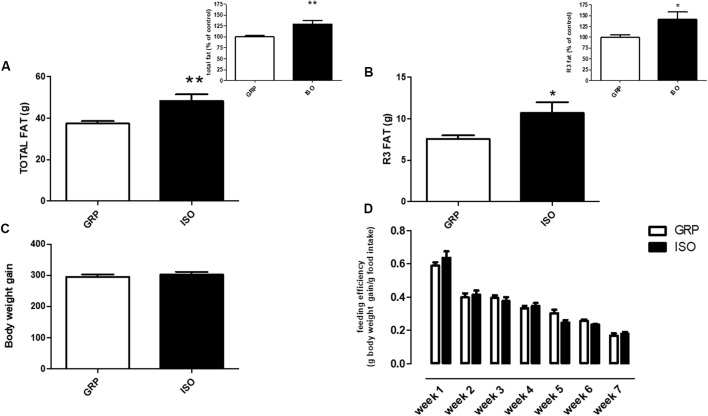
Quantification of total and visceral fat by DEXA. **(A)** Total fat quantification by DEXA in control (GRP, *n* = 10) and 7 week isolated rats (ISO, *n* = 8). Student’s *t*-test, ^∗∗^*p* < 0.01. **(B)** Visceral fat (R3) quantification by DEXA in control (GRP) and 7 week isolated rats (ISO). *n* = 10 per group, Student’s *t*-test, ^∗^*p* < 0.05. **(C)** Body weight gain quantification in control (GRP, *n* = 10) and 7 week isolated rats (ISO, *n* = 8). Student’s *t*-test, *p* = 0.009. **(D)** Feeding efficiency expressed as g body weight gain/g food intake in control (GRP, *n* = 10) and 7 week isolated rats (ISO, *n* = 8). Two-way ANOVA for time differences (*F*_1,18_ = 1.511, *p* < 0.0001; two-way ANOVA for group differences *F*_1,18_ = 0.0272, *p* > 0.05).

In order to evaluate if social isolation rearing might affect adipose tissue amount, we evaluated total and visceral fat mass by DEXA in rats reared in group and in animals exposed to a 7-week period of social isolation. We detected an increase of both total fat mass (29%) (**Figure 2A** and related inset, *n* = 10 controls and *n* = 8 isolated animals, Student’s *t*-test Welch’s correction, *p* < 0.01) and R3 fat mass (41%) (**Figure 2B** and related inset, *n* = 10 controls and *n* = 8 isolated animals, Student’s *t*-test with Welch’s correction, *p* < 0.05) in isolated rats with respect to control animals.

### Absence of Differences in Body Weight and Food Intake between Control and Isolated Animals

In order to assess if social isolation could induce any significant differences in body weight gain and food intake between control and isolated animals, we weekly measured these two parameters. At the beginning of the study (week 1 of the social isolation protocol) no difference was present between grouped and isolated rats (data not shown). As regard body weight gain, we found that the increase of body weight in isolated animals was comparable to control animals (**Figure 2C**, *n* = 10 controls and *n* = 8 isolated animals, *p* = 0.009). Furthermore, we evaluated feeding efficiency between groups. A statistical difference in this parameter was evident over time (**Figure 2D**, two-way ANOVA *F*_1,18_ = 1.511, *p* < 0.0001), as physiologically expected, while no difference was evident after between group analyses (**Figure 2D**, two-way ANOVA *F*_1,18_ = 0.0272, *p* > 0.05).

### Induction of Redox Disequilibrium in Visceral Fat after 7 Weeks of Social Isolation

**FIGURE 3:**
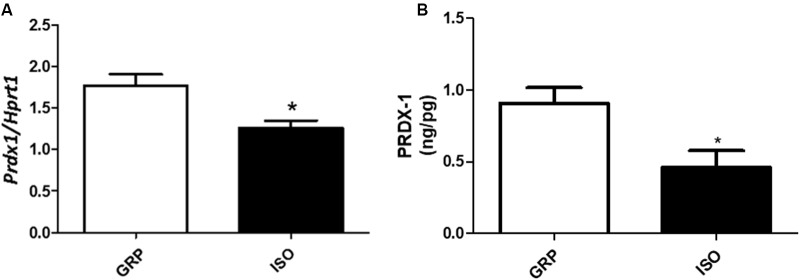
Quantification of Prdx1 (mRNA and protein) and Ucp1 (mRNA). **(A)** Quantification of Prdx1 mRNA levels by qPCR, normalized with Hrpt1 as housekeeping gene in visceral fat of control (GRP, *n* = 4) and 7 week isolated rats (ISO, *n* = 4). Student’s *t*-test, ^∗^*p* < 0.05. **(B)** Quantification of Prdx1 protein levels by ELISA (ng/pg) in visceral fat of control (GRP, *n* = 5) and 7 week isolated rats (ISO, *n* = 6). Student’s *t*-test, ^∗^*p* < 0.05.

In order to analyze the possible association between increased visceral fat amount and redox disequilibrium in isolated animals, we performed qPCR for specific genes encoding for antioxidant enzymes and ROS producers in visceral fat. We detected a decreased mRNA expression of *Prdx1*, known to be a crucial component of the antioxidant defense (Aeby et al., 2016) reducing hydrogen peroxide and alkyling hydroperoxides, in isolated animals with respect to rats which were reared in group (**Figure 3A**, *n* = 4 controls and 4 isolated animals, Student’s *t*-test, *p* < 0.05), which was also translated in a decreased expression of the corresponding protein level (**Figure 3B**, *n* = 5 controls and 6 isolated animals, Student’s *t*-test, *p* < 0.05). This was associated with decreased mRNA levels of *Ucp-1*, described as a fine regulator of the antioxidant capacity in the white adipose tissue (Jankovic et al., 2015b) (**Table 1**, *n* = 4 controls and 6 isolated animals, Student’s *t*-test, *p* < 0.05).

**Table 1:** mRNA expression of *Ucp-1, Cidea, Slc2a4*, and *Acacb* in visceral fat of controls (GRP) and isolated (ISO) rats.

Gene	GRP (*Gene*/Hprt1)	ISO (*Gene*/Hprt1)	*p*-Value
*Ucp-1*	5.985 ± 1.847	1.744 ± 0.7966	<0.05
*Cidea*	0.6350 ± 0.03958	1.197 ± 0.05485	<0.001
*Slc2a4*	0.8913 ± 0.03816	1.182 ± 0.05071	<0.01
*Acacb*	0.9998 ± 0.07958	1.585 ± 0.1864	<0.05

**FIGURE 4:**
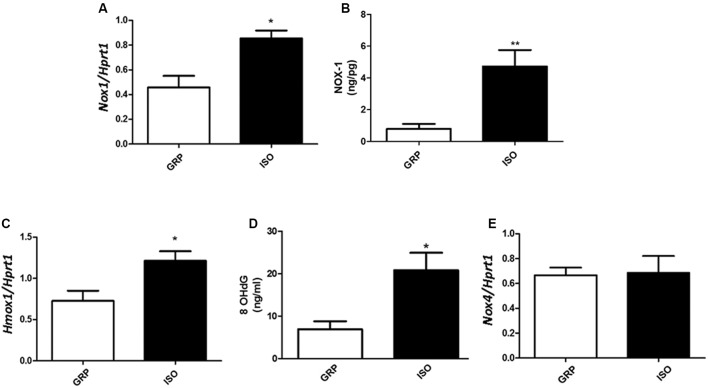
Quantification of Nox1 (mRNA and protein), Hmox1 and Nox4 mRNA and of 8OhDG. **(A)** Quantification of Nox1 mRNA levels by qPCR, normalized with Hrpt1 as housekeeping gene in visceral fat of control (GRP, *n* = 4) and 7 week isolated rats (ISO, *n* = 4). Student’s *t*-test, ^∗^*p* < 0.05. **(B)** Quantification of Nox1 protein levels by ELISA (ng/pg) in visceral fat of control (GRP, *n* = 5) and 7 week isolated rats (ISO, *n* = 6). Student’s *t*-test, ^∗∗^*p* < 0.01. **(C)** Quantification of Hmox1 mRNA levels by qPCR, normalized with Hrpt1 as housekeeping gene in visceral fat of control (GRP, *n* = 5) and 7 week isolated rats (ISO, *n* = 5). Student’s *t*-test, ^∗^*p* < 0.05. **(D)** Quantification of 8OhDG levels by ELISA in visceral fat of control (GRP, *n* = 4) and 7 week isolated rats (ISO, *n* = 5). Student’s *t*-test, ^∗^*p* < 0.05. **(E)** Quantification of Nox4 mRNA levels by qPCR, normalized with Hrpt1 as housekeeping gene in visceral fat of control (GRP, *n* = 5) and 7 week isolated rats (ISO, *n* = 6). Student’s *t*-test, *p* = 0.9101.

An enhancement in the expression of free radical producers was also observed after 7 weeks of social isolation, in terms of increased *Nox1*, a NADPH oxidase isoform implicated in ROS production in adipose tissue (Matsuzawa-Nagata et al., 2008), at both mRNA and protein levels (**Figure 4A**, *n* = 4 controls and 4 isolated animals, Student’s *t*-test, *p* < 0.05; **Figure 4B**, *n* = 5 controls and 6 isolated animals, Student’s *t*-test, *p* < 0.01), as well as mRNA of *Hmox-1* (**Figure 4C**, *n* = 5 controls and 5 isolated animals, Student’s *t*-test, *p* < 0.05), considered one of the most sensitive and reliable indicators of cellular oxidative stress and redox regulator (Poss and Tonegawa, 1997; Ryter and Choi, 2005). The observed disequilibrium between antioxidant defense and expression of ROS producers in visceral fat was further confirmed by a significant increase, in the same tissue, of 8OHdG, one of the most abundant marker of oxidative stress to DNA (Breen and Murphy, 1995), after 7 weeks of social isolation (**Figure 4D**, *n* = 4 controls and 5 isolated animals, Student’s *t*-test, *p* < 0.05). Seven weeks of social isolation did not increase *Nox4* mRNA expression in socially isolated rats compared to controls (**Figure 4E**, *n* = 5 controls and 6 isolated animals, Student’s *t*-test, *p* = 0.9101).

### Oxidative Stress-Induced Damage in Visceral Fat after 7 Weeks of Social Isolation

In order to assess if social isolation-induced increased oxidative stress in visceral fat resulted in a molecular damage of this tissue, we quantified mRNA expression of genes whose increase has been related to adipose tissue dysfunctions, i.e., *Cidea*, *Slc2a4, and Acacb* (Ma et al., 2011; Wu et al., 2014; Feldo et al., 2015; Malodobra-Mazur et al., 2016). Seven weeks of social isolation caused a significant increase in *Cidea* mRNA levels (**Table 1**, *n* = 4 controls and 5 isolated animals, Student’s *t*-test, *p* < 0.001), as well as *Slc2a4* (**Table 1**, *n* = 4 controls and 6 isolated animals, Student’s *t*-test, *p* < 0.01) and *Acacb* expression (**Table 1**, *n* = 4 controls and 5 isolated animals, Student’s *t*-test, *p* < 0.05) with respect to the rearing in non-isolation conditions.

### Oxidative Stress-Induced Impairment of Adrenergic Beta-3 Receptors in Visceral Fat after 7 Weeks of Social Isolation

**FIGURE 5:**
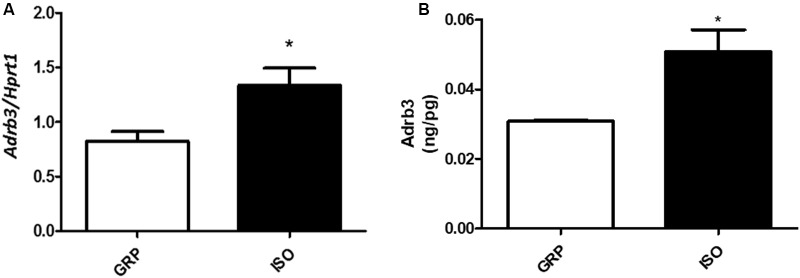
Quantification of Adrb3 (mRNA and protein). **(A)** Quantification of Adrb3 mRNA levels by qPCR, normalized with Hrpt1 as housekeeping gene in visceral fat of control (GRP, *n* = 4) and 7 week isolated rats (ISO, *n* = 4). Student’s *t*-test, ^∗^*p* < 0.05. **(B)** Quantification of Adrb3 protein levels by ELISA (ng/pg) in visceral fat of control (GRP, *n* = 5) and 7 week isolated rats (ISO, *n* = 6). Student’s *t*-test, ^∗^*p* < 0.05.

In order to determine the impact of social isolation-induced increased oxidative stress in visceral fat and adrenergic beta receptors, we performed Real-Time PCR for the subtype 3. We found a significant elevation in the mRNA levels of *Adrb3* in visceral fat derived from 7 week isolated animals compared to controls (**Figure 5A**, *n* = 4 controls and 4 isolated animals, Student’s *t*-test, *p* < 0.05). This was accompanied by a significant elevation of Adrb3 protein levels (**Figure 5B**, *n* = 5 controls and 6 isolated animals, *p* < 0.05).

## Discussion

In this study, we investigated alterations of total and visceral fat amount in a non-pharmacologic rodent model of psychosocial-stress induced psychosis, obtained by rearing rats in social isolation for 7 weeks. We observed a significantly increased amount of both total and visceral fat in socially isolated rats with respect to animals reared in group. However, despite the observed adipose tissue dysfunctions, isolated animals, at least in our experimental conditions, were not obese, as their body weight gain and food intake efficiency were not significantly different from the ones of control rats. While psychotic disorders have been widely associated with metabolic disturbances, as well as adipose tissue dysfunctions (Seeman, 2014; Nenke et al., 2015), this has been mostly attributed to the impact of the antipsychotic therapy on weight, body, mass index, and adipocyte functions. Indeed, together with the well-described effects of typical neuroleptics on fat mass and distribution (Panariello et al., 2011), increased adipose tissue amount, alterations of fat body composition, as well as changes in adipose mass distribution have also been described following treatment with atypical antipsychotics (Konarzewska et al., 2014), especially following olanzapine treatment (Chintoh et al., 2008; Mann et al., 2013). In this context, the present work shows novel aspects with respect to the existing literature in the field. First, very poor data are actually available on the possible impact of the psychotic disorder *per se* on adipose tissue amount, distribution and functioning, as most of preclinical data have been obtained by using pharmacological animal models of this disorder, such as the ketamine model (Coccurello and Moles, 2010). Moreover, although some clinical studies have described weight elevation, adipose tissue dysfunctions, as well as other metabolic alterations, in drug-free and drug-naive psychotic patients (Ryan et al., 2003, 2004; Spelman et al., 2007), limited evidence still exist on this subject, mostly because some existing reports on the pre-medication era present some important biases in the methodological aspects and in the definition of the concept of “drug-free” and “drug-naïve” patients. Indeed, some studies have included, as “drug-free,” patients medicated for a short period of time (for example receiving therapy for up to 30 days prior to investigations) or receiving an anti-anxiety medication, such as lorazepam, the night before blood was drawn for analysis (Mane et al., 2009). Thus, the present study represents the first pre-clinical *in vivo* evidence highlighting a pathogenetic link between psychosis *per se* and adipose tissue impairment, without any kind of possible pharmacological interference. Indeed, the animal model of psychosis used in the present study is totally “drug-free,” being obtained by exposing rats to an environmental psychosocial stressor, the rearing in isolation conditions for a period of 7 weeks (Weiss and Feldon, 2001; Leng et al., 2004; Fone and Porkess, 2008). Some previous publications reported the effects of social isolation on body weight and other markers of fat dysfunctions (Yamada et al., 2000, 2015; Nonogaki et al., 2007; Ryu et al., 2009; Krolow et al., 2013). However, there are several important differences between these previous works and ours, in terms of used rodent species, social isolation procedure performing, time of the isolation period and presence of metabolic comorbidities. The observed increase in visceral fat in isolated animals is in line with several previous pre-clinical and clinical studies reporting a direct pathogenetic link between psychosocial stress and visceral fat elevation. In particular, some investigations, performed by using rodents under chronic psychosocial stress, highlighted the development of metabolic alterations, energy imbalance and adipose tissue impairment (Moles et al., 2006; Bartolomucci et al., 2009). Interestingly, a recent clinical study also described a link between enhanced abdominal adiposity and exposure to psychosocial stressors in youth (Shearrer et al., 2016).

In this regard, another novelty of the present study is represented by our findings highlighting a crucial role of redox imbalance in visceral fat of rats following 7 weeks of social isolation. Indeed, decreased mRNA levels of *Prdx1* and *Ucp-1*, two important players of the antioxidant defense, were detected in isolated rats with respect to animals reared in group. *Prdx1* is a member of the peroxiredoxin family of antioxidant enzymes, reducing hydrogen peroxide and alkyl hydroperoxides (He et al., 2015), involved in the maintenance of different physiological functions, as well as in the pathogenesis of several diseases, such as inflammation-mediated disorders, tumorigenesis (El Eter and Al-Masri, 2015) and neurological illnesses (Zhu et al., 2012). Interestingly, a decrease in *Prdx1* has been also associated with the development of impaired metabolism-associated disorders, such as type 2 diabetes and cardiovascular diseases (El Eter and Al-Masri, 2015). On the other hand, *Ucp-1*, beyond its well-known physiological role in energy wasting in the brown adipose tissue (Porter, 2006), has been described as an important player of the antioxidant defense specifically implicated in the protection of the white adipose tissue against the increased free radical production induced by different conditions, such as cold exposure (Jankovic et al., 2015a). The observed decreased antioxidant capacity detected in the visceral fat of isolated rats is in line with previous reports describing depletion of antioxidant enzymes, such as superoxide dismutase, catalase, and glutathione peroxidase in case of adipose tissue dysfunctions and obesity characterized by visceral fat accumulation (Ozata et al., 2002; Fernandez-Sanchez et al., 2011). Interestingly, some studies described a lower liver concentration of vitamin A in rats with increased visceral fat mass compared with controls (Capel and Dorrell, 1984) as well as decreased serum levels of other antioxidants, such as vitamin E, vitamin C, and β-carotene (Vincent et al., 2005). Moreover, it has been widely demonstrated that exposure to chronic psychosocial stress leads to decreased antioxidant enzyme expression and functioning both in animal models and humans (Eskiocak et al., 2005; Djordjevic et al., 2010; Tsuber et al., 2014). A crucial role of increased ROS production has been described in the development of psychosocial stress induced disorders in both rodents and humans. In this context, a novelty of the present manuscript with respect to our previous publications, showing a crucial role of the NADPH oxidase Nox2-derived oxidative stress in the pathogenesis of neuropathological alterations induced by rat social isolation (Schiavone et al., 2009, 2012, 2017) and in the onset and progression of isolation-induced HPA-axis alterations (Colaianna et al., 2013), is related to our findings demonstrating an increased expression of ROS producing enzymes in visceral fat of socially isolated rats compared to animals reared in group. More specifically, we observed an enhanced expression of the NADPH oxidase Nox1 enzyme in visceral fat after 7 weeks of social isolation. In line with this observation, ROS production in mice with increased visceral fat was attributed to an augmented expression of NADPH oxidase (Furukawa et al., 2004). In addition, in mice with increased visceral fat mass, treatment with NADPH oxidase inhibitors was shown to reduce ROS production in adipose tissue and to attenuate excessive visceral fat-induced metabolic alterations (Furukawa et al., 2004). Interestingly, at least in our experimental conditions, we did not observe any increase of the NADPH oxidase Nox4. This enzyme has been shown to play crucial physiological roles in adipose tissue, such as the differentiation and proliferation of pre-adipocytes (Schroder et al., 2009) as well as the insulin response (Mouche et al., 2007). Although some studies point toward a crucial role of Nox4 enzymes in adipose tissue dysfunctions, as well as metabolic disorders (Le Lay et al., 2014), this has been mainly related to the weight gain associated with this conditions (Jiang et al., 2011). Importantly, in this work, we showed the lack of significant differences in body weight gain between isolated and grouped animals, confirming what previously reported (Schiavone et al., 2016). Hence, this could account for the absence of Nox4 alterations in the visceral fat of isolated animals. Moreover, a recent work reported that ROS overproduction in abdominal fat-derived mesenchymal stromal cells was accompanied by increased expression of Nox1 but not of Nox4 (Sela et al., 2015). In the present study, we demonstrated that 7 weeks of social isolation lead to increased *Hmox-1* mRNA levels. ROS production and redox imbalance have been related to enhanced *Hmox-1* expression (Nath et al., 2001; Ulyanova et al., 2001). Importantly, elevations in the levels of this enzyme have been observed in peripheral alterations induced by chronic psychosocial stress in mice (Czech et al., 2013) and, in particular, in adipose tissue dysfunctions and metabolic disorders (Lohr et al., 2015). Further on, *Hmox-1* expression and functioning have been demonstrated to be strictly dependent from NADPH oxidase Nox enzymes (Rousset et al., 2013), especially during fat mass function regulation (Depner et al., 2013).

We also demonstrated that visceral fat increased oxidative stress was accompanied by adipose tissue damage, assessed by *Cidea* and *Acacb* mRNA expression. Our observations are supported by previous studies reporting an association between the overexpression of these genes and oxidative stress-related detrimental processes occurring in visceral fat (Ma et al., 2011; Wu et al., 2014; Feldo et al., 2015; Malodobra-Mazur et al., 2016). Furthermore, our results showing *Slc2a4* overexpression in rodent white adipose tissue are in line with previous observations reporting an increased expression of this gene at the very early stage of obesity (Malodobra-Mazur et al., 2016). Indeed, although the social isolation procedure we applied did not induce obesity, at least in terms of increased body weight and food intake efficiency, it cannot be excluded that the detected adipose tissue dysfunctions might represent a very early stage of this disorder.

In this study, we showed increased mRNA levels of *Adrb3* in 7 week isolated rats with respect to grouped animals. These receptors have been known to play important physiologic and pathological functions in visceral fat since long time (Lowell and Flier, 1995; Lipworth, 1996; Coman et al., 2009). In particular, it has been shown that the adrenergic beta receptor system is highly dysfunctional in adipose tissue disorders and the ability to regulate both lipolysis and thermogenesis is also significantly impaired (Collins and Surwit, 2001). Importantly, several studies reported a strong association between increased expression of *Adrb3* and elevations in ROS production in different body districts, such as the cardiovascular and the CNS (Black, 2015; Karimi Galougahi et al., 2016; Yoshioka et al., 2016). Interestingly, a possible molecular link between enhanced adrenergic beta 3 receptor levels and increased free radical amount has been referred to an increased expression and activation of the NADPH oxidase system (Karimi Galougahi et al., 2015, 2016). In particular, some findings highlighted that β-adrenergic antagonists may have a role in cell proliferation by suppressing NADPH oxidase Nox1 and Nox2 (Kobayashi et al., 2003; Mollnau et al., 2003; Manrique et al., 2011; Sorrentino et al., 2011). Our finding of increased *Adrb3* in this animal model of psychosocial stress-induced psychosis represents a significant novel aspect of this work, as available studies in literature are mainly focused on the possible impact of the antipsychotic medication on these receptors (Tsai et al., 2004; Ujike et al., 2008), while no data on the link between psychosis *per se* and *Adrb3* exist neither in rodents or humans. Thus, our study provides the first pre-clinical *in vivo* evidence on the existence of increased *Adrb3* independently from antipsychotic medication.

Based on the obtained evidence, the following molecular mechanism could be hypothesized: exposure to chronic stress, represented by 7 weeks of social isolation, might disrupt the physiological redox balance, both decreasing the expression and most likely the functioning of antioxidant enzymes and increasing the production of free radicals by specific enzymatic systems, such as the NADPH oxidase, in visceral fat. The consequent elevation in ROS production might account for adipose tissue dysfunctions, including alterations of *Adbr3* at both mRNA and protein levels, possibly in association with other peripheral detrimental processes mediated by isolation-induced inflammatory pathways and HPA-axis functioning disturbances (Colaianna et al., 2013).

A possible limitation in the interpretation of our results could be represented by the fact that the social isolation model has been also used as a tool to induce depression or anxiety in animals and that these disorders, especially depression, have been shown to be related *per se* to disturbances in patient eating behavior. Thus, although in our isolated animals we did not observe any disturbances of the eating behavior, further research are needed in other animal experimental models of schizophrenia to evaluate the persistence of the effect on fat mass across them.

Importantly, the role of redox imbalance in contributing to visceral fat dysfunctions in isolated animals could be considered also crucial if extrapolated to humans suffering from psychosis, given the well-known impact of both oxidative stress and adipose tissue dysfunctions in the cardiovascular risk. Moreover, another important translational approach toward the human pathology would be the possibility to consider the cardiovascular risk in schizophrenic patients independent from the fat dysfunctions derived from medication rather than associating this risk to oxidative stress and consequent fat dysfunctions due to psychosis *per se*. In conclusion, our data suggest that 7 weeks of social isolation causes visceral fat accumulation and redox imbalance in this tissue, probably mediated by decreased antioxidant defense as well as enhanced NADPH oxidase expression, most likely leading to oxidative-stress related damage and increased *Adrb3* pathways. In this context, the pharmacological selective targeting of specific Nox enzymes as well as the enhancement of the antioxidant capacity might represent innovative approaches for the treatment of peripheral alterations associated with psychosis.

## Author Contributions

Study design: SS, MGM, and LT; study conduct: GMC, EM, MZ, MC, EC and MB; data collection: PT, ADG, EM, and AT; data analysis: SS and MGM; data interpretation: SS, MGM, and LT; drafting manuscript: SS and LT; revising manuscript content: SS, GMC, EM, MZ, MC, ADG, AT, FPC, EC, MB, PT, MGM, and LT; approving final version of the manuscript: SS, GMC, EM, MZ, MC, ADG, AT, FPC, EC, MB, PT, MGM, and LT; LT takes responsibility for the integrity of the data analysis.

## Conflict of Interest Statement

The authors declare that the research was conducted in the absence of any commercial or financial relationships that could be construed as a potential conflict of interest.
